# Cognitive Stimulation Induces Differential Gene Expression in *Octopus vulgaris*: The Key Role of Protocadherins

**DOI:** 10.3390/biology9080196

**Published:** 2020-07-30

**Authors:** Valeria Maselli, Gianluca Polese, Al-Sayed Al-Soudy, Maria Buglione, Anna Di Cosmo

**Affiliations:** Department of Biology, University of Napoli Federico II, Complesso Universitario Monte Sant’Angelo, Via Cinthia, Build 7, 80126 Napoli, Italy; valeria.maselli@unina.it (V.M.); gianluca.polese@unina.it (G.P.); alsayedalsoudymohamed.mostafa@unina.it (A.-S.A.-S.); maria.buglione@unina.it (M.B.)

**Keywords:** octopus, lophotrochozoan brain, acclimatization experiment, cognitive stimulation, adult neurogenesis gene markers, PCDHs, cephalopods

## Abstract

Octopuses are unique invertebrates, with sophisticated and flexible behaviors controlled by a high degree of brain plasticity, learning, and memory. Moreover, in *Octopus vulgaris*, it has been demonstrated that animals housed in an enriched environment show adult neurogenesis in specific brain areas. Firstly, we evaluated the optimal acclimatization period needed for an *O. vulgaris* before starting a cognitive stimulation experiment. Subsequently, we analyzed differential gene expression in specific brain areas in adult animals kept in *tested* (enriched environment), *wild* (naturally enriched environment), and *control* conditions (unenriched environment). We selected and sequenced three protocadherin genes (PCDHs) involved in the development and maintenance of the nervous system; three Pax genes that control cell specification and tissue differentiation; the Elav gene, an earliest marker for neural cells; and the Zic1 gene, involved in early neural formation in the brain. In this paper, we evaluated gene expression levels in *O. vulgaris* under different cognitive stimulations. Our data shows that Oct-PCDHs genes are upregulated in the learning and lower motor centers in the brain of both tested and wild animals (higher in the latter). Combining these results with our previous studies on *O. vulgaris* neurogenesis, we proposed that PCDH genes may be involved in adult neurogenesis processes, and related with their cognitive abilities.

## 1. Introduction

Octopuses have considerable skills that show them as “intelligent” animals. They express high flexibility in solving demanding problems, and they have been observed using objects as tools. Octopuses also learn very fast when faced with artificial tasks [1,2,3,4,5,6,7,8,9].

Recently, it has been observed that *Octopus vulgaris* cognition and learning abilities are also linked to adult neurogenesis [10]: Animals housed in an enriched environment increase adult neurogenesis, using Proliferating Cell Nuclear Antigen (PCNA) as a marker of cell proliferation and a cytoplasmic isoform of poli (ADP-ribose) polymerase 1 (PARP1) as a marker of neuronal plasticity [10,11]. Octopuses, subjected to problem-solving tasks, revealed an increment of cell proliferation in supraesophageal mass, in particular in the vertical-frontal system and the optic–olfactory lobes, brain areas involved in learning memory, and sensory stimuli integration, respectively [10]. Moreover, bivariant analysis of flow cytometry using BrdU incorporation allowed assessment of the magnitude of adult neurogenesis in those brain lobes, previously identified, characterized by the presence of adult neurogenesis niches, highlighting the amount of cells exhibiting de novo DNA synthesis [12]. What remains puzzling is what genes are majorly involved in these intriguing processes in *Octopus vulgaris*.

In cephalopods, assembled transcriptomes reveal a substantial expansion of protocadherin genes (PCDHs) except for *Nautilus*, which lacks the elaborated Coleoid nervous system [13,14]. The genetic divergence between octopus and squid PCDHs expansions may reflect the notable differences between octopuses and decapodiformes in brain organization. PCDH genes are orthologous in *O. vulgaris* and *O. bimaculoides*, suggesting that the PCDHs’ expansion occurred before the speciation [15]. Strikingly, the octopus and squid PCDHs are significantly enriched with RNA editing sites, especially in *O. vulgaris* [14].

The octopus genome encodes 168 multi-exonic PCDH genes, nearly three-quarters of which are found in tandem clusters on the genome, which is 10 times more than many vertebrates, and more than twice as many as humans and other mammals. PCDHs are expressed in adult mammalian brains, especially in the hippocampus, cerebellum, and cortex [16,17,18,19,20], suggesting a role in adult brain functioning, beyond the establishment of neural connectivity [21,22]. The expression of PCDHs in octopus’s neural tissues and the high number of editing sites are consistent with a central role for these genes in development and maintenance of coleoids’ nervous system organization as they do in vertebrates [13,14].

Additionally, Pax genes encode for a family of metazoan transcription factors, which are essential for cell specification and tissue differentiation, including the nervous system [23,24,25,26]. In vertebrates, *pax3* and *pax7* contribute to the development of the nervous system [27,28,29]; their homologs in *Drosophila* [30,31] are essential segment-polarity genes [32] and are involved in neurogenesis [33]. In the Lophotrochozoa studied so far, Platyhelmintha [34,35], Annelida [36], Mollusca [26,37,38,39,40,41], Brachiopoda [42], and Nemertea [43], the expression patterns of each Pax gene suggest conserved and consistent roles for *pax*3/7, *pax*2/5/8, and *pax6* in the nervous system development, in the sensory structure formation, and in the eye morphogenesis, respectively. In particular, among Cephalopods, in *Sepia officinalis*, *Sof-pax6* expression was largely distributed in the central nervous system (CNS) and in the brachial nervous chord [44]. Likewise, the restriction of *Sof-pax6* expression at the distal tip of growing arms, which is described as a growing/proliferation region in *Octopus* [45] and *Euprymna* [46], suggests a role of *pax6* in the promoting proliferation mechanisms underlying the growth of the arms. Furthermore *S*of-*pax3/7* neural expression occurs later, suggesting that these genes are not involved in early neurogenesis, but it is restricted to the ventral brain in “motor” areas controlling the arms [44]. Moreover, *Sof-pax2/5/8* might be involved in early steps of locomotor structures’ development derived from the ancestor mollusk foot [44]. They could be implicated in the formation of the whole nervous circuitry controlling arms and funnel muscles [44].

In annelid and *Drosophila*, ELAV is the earliest marker for neural cells as they just exit the cell cycle and start to differentiate into neurons [47,48]. The ELAV protein is present exclusively in all immature and mature neurons [49]. In addition, its upregulation in rodent hippocampal neurons, having learned a spatial discrimination paradigm, suggests a role in memory storage [50]. In *Drosophila*, ELAV is necessary for neuronal differentiation [51], and it is expressed pan-neurally in all stages of development. In *Sepia*, *Sof-elav* appears to be involved in neurogenesis during embryogenesis [52].

ZIC family members, instead, play key roles in early neural patterning and the development of the neural crest, visual system, and cerebellum in mammals [53,54]. *zic* homologs have been identified in chordates, arthropods, and nematodes [55]. Moreover, *zic* family genes are expressed in a subset of the developing brain and mesoderm derivatives in annelids [55].

In *Octopus vulgaris*, many genes have been hypothesized to have a role in its cognitive abilities, but up to date, their roles have been suggested just based on genomic and transcriptomic analysis [13]. Nerveless, it has never been demonstrated if and how their expressions fluctuate after cognitive stimulations.

We than sequenced two isoforms of Oct-PCDH18 and one of Oct-PCDH15. The two isoforms of PCDH18 found in zebrafish play a crucial role in the brain: PCDH18a has a role in cell adhesion and migration [56], whereas PCDH18b interacts with Nap1, an important regulator of actin dynamics, to control motor axon growth and arborization in primary motoneurons [57]. PCDH15 expression was described in the adult brain in mice [17], and it is expressed in inner ear hair cell stereocilia and retinal photoreceptors in humans [58].

As potential co-actors of PCDHs in octopus cognitive processes, we sequenced *Oct-pax2/5/8*, *Oct-pax3/7*, *Oct-pax6, Oct-elav*, and *Oct-zic1*. We then analyzed for all these selected genes their differential expression patterns under different behavioral cognitive stimulations.

## 2. Materials and Methods

### 2.1. Experimental Model and Subject Details

*Octopus vulgaris* specimens (female n = 33, weight 800 ± 50 g), collected in Bay of Naples, were transferred to the Department of Biology [59].

Animals were housed in individual tanks to prevent aggressive social interactions and cannibalism. They were housed in PVC tanks (50 × 50 × 50 cm), covered with a Plexiglas lid to avoid animals’ escape, equipped with a den, natural sand, and shells. Water and room temperature were maintained at 18 °C, and the light/dark cycle was set to the natural photoperiod. Water was treated with biological filters and protein skimmers. First days of captivity were considered as the acclimatization period, during which several physiological and behavioral parameters were monitored to verify the welfare and healthiness of the octopuses [59,60]. During the acclimatization phase, animals were fed by experimenters with their natural prey: Crabs (*Carcinus mediterraneus*) or mussels (*Mytilus galloprovincialis*) once a day.

**Figure 1:**
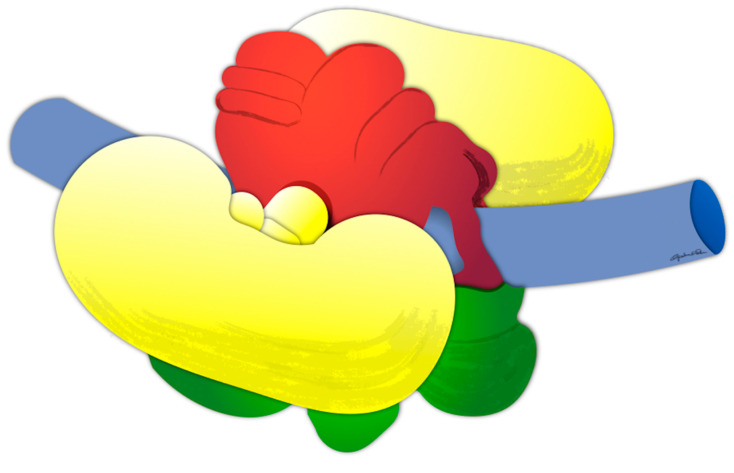
Schematic view of *Octopus vulgaris* brain anatomy, highlighting the tissues sampled for analysis: the optic lobes with olfactory and peduncle lobes on the optic tracts (OOP, yellow), the central part of the supraesophageal mass (red), and the subesophageal mass (green) that enwraps the esophagus (blue, not analyzed).

Octopuses were anaesthetized by isoflurane insufflation [61] and brains were dissected in sterile conditions, isolating the central part of the supraesophageal mass, the subesophageal mass, and the optic lobes including olfactory and peduncle lobes on the optic tracts (OOP, Figure 1). Dissected samples were stored at −80 °C for further experiments.

Our research conformed to European Directive 2010/63 EU L276, the Italian DL. 4/03/2014, no. 26, and the ethical principles of Reduction, Refinement, and Replacement (Project n° 608/2016-PR- 17/06/2016; protocol n° DGSAF 0022292-P-03/10/2017).

### 2.2. Acclimatization Experiment

In order to experimentally quantify the acclimatization time required for octopuses, we performed an initial experiment to determine the time needed by octopuses to feel comfortable and ready to face the challenge of opening two jars containing food.

Octopuses (N = 3 for 5 groups, total N = 15) were tested at different times after they arrived in the lab for 3 consecutive days, once a day, with two plastic jars closed with a screw lid containing a live prey. During experimental days, octopuses had no feeding opportunities except to open a jar. The first group was tested since day 1, the second starting on day 2, the third on day 6, the fourth on day 14, and the fifth on day 19, measuring the average of opened jars during the training period. Jar position was constant through the experiments, and behavioral quantification was restricted to measuring the average number of opened jars.

Following the results of the acclimatization experiment, we set up a period 14 days for the next experiments.

### 2.3. Cognitive Stimulation

Novel animals (N = 18) were used in three experimental groups: Control, tested, and wild. The control animal group (N = 6) was not tested, and they were fed regularly without any task for 17 days (14 acclimatization + 3 experimental days). The tested animal group (N = 6) was tested for the consecutive 3 days after the acclimatization period (14 days).

We altered the standard housing conditions providing a cognitive challenge. During 3 experimental days, once a day, octopuses were tested with two jars containing a live prey and closed with a screw lid. During experimental days, octopuses had no feeding opportunities except to open the jars to reach the prey [10]. The wild animal group (N = 6) was captured and directly sacrificed.

All experiments (acclimatization and cognitive stimulation) were conducted once per day and recorded for at least 1 h with a digital camera (GoPro Hero 5, GoPro, Inc. CA, USA) positioned on the front of the aquarium (20 cm), to analyze the octopus’s choice and behavioral responses, such as exploring, opening the jar, and eating.

### 2.4. RNA Extraction, Selection and Primer Design, and Gene Expression Pattern

Total RNA was extracted using the RNeasy minikit (Qiagen, Valencia, CA, USA). The quality and amount of purified RNA were analyzed spectrophotometrically with Qubit 3.0 (Thermo Scientific Inc., Waltham, MA, USA). RNA of 1000 ng was reverse transcribed with the QuantiTect^®^ Reverse Transcription Kit (Qiagen). Three members of the PCDH family (PCDH15, PCDH18a, and PCDH18b), pax2/5/8, pax3/7, pax6, elav, and zic1 were characterized in an EST library and initial amplification primers for polymerase chain reaction (PCR) were synthesized using the software Geneious 9.1 (Table S1). PCRs were performed in a final volume of 20 μL, with 0.2 μL of Pfu DNA polymerase (Thermo Scientific), 4 μL of 4× Tris buffer with MgCl2, 1.6 μL of dNTPs (each dNTP 2.5 μM), 0.2 μL of 50 μM of each primer, and 100 ng of cDNA template under the following conditions: An initial denaturing step of 98 °C for 3 min; 35 cycles of 10 s at 98 °C; 30 s at 55–60 °C and 1 min at 72 °C; and a final extension step of 5 min at 72 °C. PCR products were purified from unincorporated primers using Exonuclease I and Fast Alkaline Phosphatase (Thermo Scientific). The sequencing reaction was performed using the BigDyeTM Terminator Cycle Sequencing chemistry (Applied Biosystems, Foster City, CA, USA). Sequences were purified using AutoSeq G-50 (Amersham, Uppsala, Sweden) spin columns and analyzed by an ABI 3100 automated sequencing instrument (Perkin-Elmer, Genetic Analyzer, Foster City, CA, USA). Chromatograms were assembled and analyzed using software Geneious version 9.1 (Biomatters, Auckland, New Zealand, available from http://www.geneious.com). PCR products were analyzed with GenBank BLASTn and BLASTx (BLAST, basic local alignment search tool). The analysis of the sequencing confirmed the identity of the fragments. All sequence data generated in this study were deposited in GenBank (accession numbers MN138036-43).

Additionally, we performed a real-time PCR using the QuantiTect SYBR Green PCR Kit (Qiagen). PCR was performed in a final volume of 25 µL, with 50 ng of cDNA, 1 mM of each primer, and 12.5 µL of QuantiFast SYBR Green PCR Master Mix (2×). The PCR cycling profile consisted of a cycle at 95 °C for 5 min, 40 three-step cycles at 95 °C for 15 s, at 60 °C for 20 s, and at 72 °C for 20 s. Quantitative RT-PCR analysis was conducted by using the 2-(ΔΔCt) method [62]. RT-PCR was performed in a Rotor-Gene Q cycler (Qiagen). Specific primers were designed (Table S1) for seven target genes and the *ubiquitin* gene was used for normalization of the relative expression. At the end of each test, a melting curve analysis was done (plate read every 0.5 °C from 55 to 95 °C) to determine the formation of the specific products. Each sample was tested and run in duplicate.

The control and the treatment groups in various assays were compared and analyzed using a Wilcoxon two group test and data with *p*-values < 0.05 were considered statistically significant.

## 3. Results

### 3.1. Acclimatization Experiment

We measured the efficiency of the acclimatization process as the average of jars opened after different numbers of days (Figure S1). The first animal group tested after the 1 day of acclimatization completely ignored the jars standing at the corner for most of the time. This happened during all 3 trial days (3TD). The second animal group that started to be tested on the second day was able to open just one jar on average during the 3TD. The third animal group was able to open 1.3 jars on average during the 3TD. The following fourth animal group opened 1.7 jars on average during the 3TD. The last animal group that started to be tested on day 19; in the following 3TD, they were able to open both jars. The number of days of acclimatization had a significant influence on the establishment of testing, as it occurred mostly on days 14–20 of the experiment, raising the level of performance in the tests after day 14 in captivity.

### 3.2. Gene Expression Patterns

#### 3.2.1. Control Animal Group

**Figure 2:**
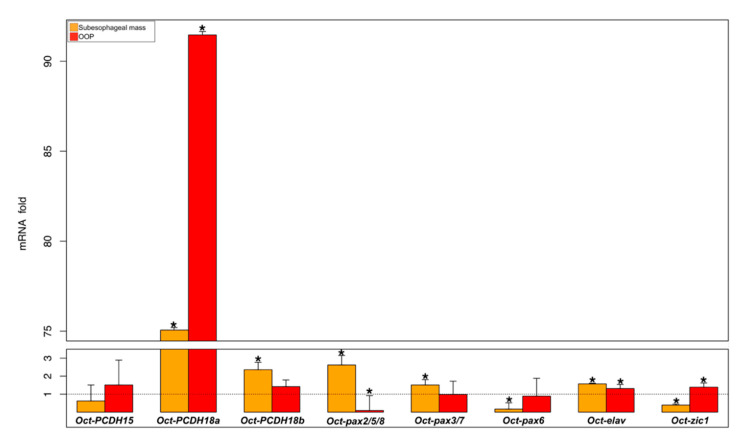
Gene expression analysis of different brain regions of the control animal group (N = 6). Relative mRNA fold change in gene expression in the subesophageal mass (orange) and OOP (red) compared to the supraesophageal mass (dot line set y = 1). * asterisk indicates that the difference vs. supraesophageal mass expression is statistically significant (Wilcoxon-test, *p* < 0.05). Error bars represent the SEM.

Of the control group animals, we evaluated the transcription activities of the selected genes (Oct-PCDH15, Oct-PCDH18a, Oct-PCDH18b, Oct-pax2/5/8, Oct-pax3/7, Oct-pax6, Oct-elav, and Oct-zic1 genes) in three brain regions, the central part of the supraesophageal mass, the subesophageal mass, and the optic lobes, including the olfactory and peduncle lobes (OOP) on the optic tracts (Table S2 and Figure 2).

The subesophageal mass, compared to the supraesophageal mass, showed a higher expression for Oct-PCDH18a and b, Oct-pax2/5/8, Oct-pax3/7, and Oct-elav genes, and lower gene expression for Oct-pax6 and Oct-zic1 genes; there were no significant differences for the Oct-PCDH15 gene (Table S2, orange bar in Figure 2). The OOP compared to the central supraesophageal mass showed a higher expression for Oct-PCDH18a, Oct-zic1, and Oct-elav genes, and a significantly lower expression was observed only for the Oct-pax2/5/8 gene (Table S2 and red bar in Figure 2). In particular, the Oct-PCDH18a gene showed a substantial increase in the subesophageal mass (about 70 times higher) and in the OOP (about 90 times higher; Table S2 and Figure 2).

#### 3.2.2. Tested and Wild Animal Groups

The tested animal group (N = 6) was acclimatized for 14 days and animals were trained for 3 consecutive days using two plastic jars. All octopuses were considered to have the same behavioral performance, responding to the test stimulation, opening both jars from the first day of the experiment, progressively decreasing the time spent opening the jar during the 3 days of training.

**Figure 3:**
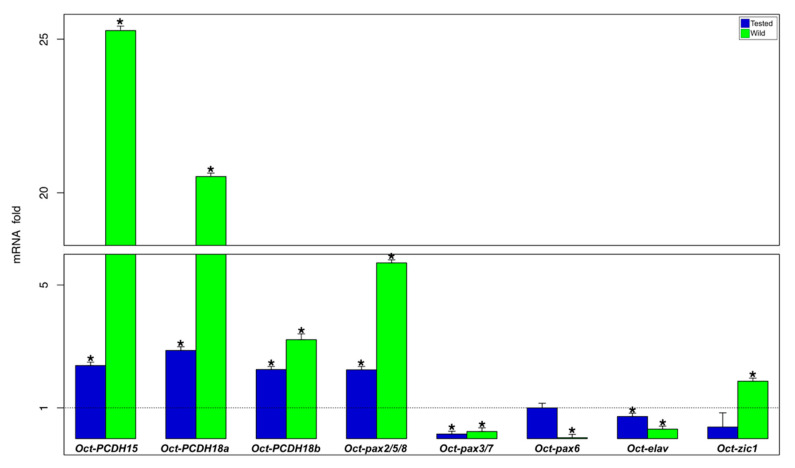
Gene expression analysis in the supraesophageal mass. Relative mRNA fold change in gene expression in tested (blue) and wild (green) animal groups are compared to the control (N = 6/group; dot line set y = 1). * asterisk indicates that the difference vs. control group is statistically significant (Wilcoxon-test, *p* < 0.05). Error bars represent the SEM.

Comparing the results among wild, tested, and control animals, it is possible to observe that in the supraesophageal mass (Table S3 and Figure 3), tested and wild animals showed an upregulation for Oct-PCDH15, Oct-PCDH18a, Oct-PCDH18b, and Oct-pax2/5/8 genes (Table S3 and Figure 3). The Oct-pax3/7 gene showed a downregulation in both tested and wild animals. Meanwhile, the Oct-zic1 gene is significantly downregulated in wild octopus. Interestingly, the Oct-elav gene in the supraesophageal mass is downregulated in tested animals and it is upregulated in wild animals (Table S3 and Figure 3).

**Figure 4:**
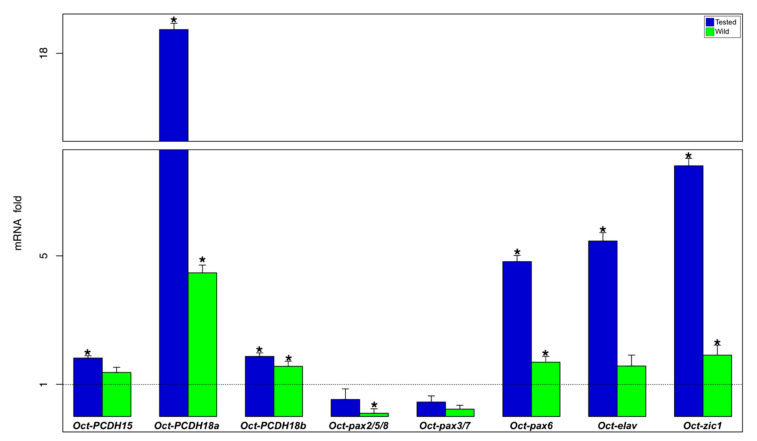
Gene expression analysis in OOP. Relative mRNA fold change in gene expression in tested (blue) and wild (green) octopus are compared to the control (N = 6/group; dot line set y = 1). * asterisk indicates that the difference vs. control group is statistically significant (Wilcoxon-test, *p* < 0.05). Error bars represent the SEM.

In the OOP, tested and wild animals showed an upregulation for Oct-PCDH18a, Oct-PCDH18b, Oct-pax6, and Oct-zic1 genes (Table S3 and Figure 4). Oct-PCDH15 and Oct-elav genes showed an upregulation in tested animals and no significant differences in the wild ones (Table S3 and Figure 4). Quite the opposite, the Oct-pax2/5/8 gene showed lower expression in wild animals and no significant difference was found in the tested ones (Table S3 and Figure 4).

**Figure 5:**
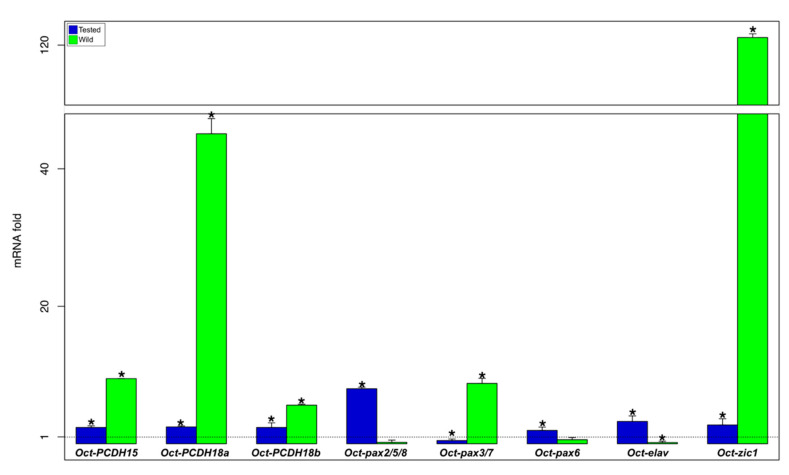
Gene expression analysis in the subesophageal mass. Relative mRNA fold change in gene expression in tested (blue) and wild (green) octopus is compared to the control (N = 6/group; dot line set y = 1). * asterisk indicates that the difference vs. control group is statistically significant (Wilcoxon-test, *p* < 0.05). Error bars represent the SEM.

In the subesophageal mass, tested animals showed a synergistic upregulation of all genes considered, except for Oct-pax3/7 (Table S3 and Figure 5). This pattern is confirmed for Oct-PCDH15, Oct-PCDH18a, Oct-PCDH18b, and Oct-zic1 genes, which showed a strong upregulation in wild animals compared not only with control animals but also with tested octopuses (Table S3 and Figure 5). Wild animals showed a significantly high level of expression also for the Oct-pax3/7 gene (Table S3 and Figure 5). Oct-elav gene expression was higher in tested animals and downregulated in wild ones (Table S3 and Figure 5). Moreover, Oct-pax2/5/8 and Oct-pax6 genes did not show significant differences in the wild octopus compared to the control animals (Table S3 and Figure 5).

## 4. Discussion

Adult neurogenesis can be considered as an intrinsic factor to the maintenance of a healthy brain and to memory assessment [63]. *O. vulgaris* possesses high cognitive capabilities, and just recently, it has never been shown that they have adult neurogenesis [10,12]. We now extend the knowledge to the genes involved in this process.

### 4.1. Selected Gene Expression Analysis in Octopus Brain Areas

Our study is the first to describe how the enhancement of the environmental conditions, in wild and tested octopuses, affects the RNA expression level in genes involved in cell proliferation and synaptogenesis, compared to control animals, that lack any environmental stimulation.

Interestingly, we observed that, among the analyzed genes possibly involved in adult neurogenesis, wild animals show higher PCDH expression either than tested or control animals. In all examined brain areas, their expression level showed a positive trend from the control, tested, and wild animal groups (Figure 5). Since environmental/cognitive stimulation enhances adult neurogenesis in *O. vulgaris* [10,12], these results strongly support the suitability of PCDHs’ expression level as a marker for this process.

### 4.2. Learning Centers of the Octopus’s Brain

The supraesophageal mass contains the vertical lobe and the superior frontal lobe (VL-SFL); this system plays an important role in learning and memory. Furthermore, the supraesophageal mass is also the site of visual learning and tactile memory, respectively localized in the vertical/subvertical/superior frontal lobes and inferior frontal lobe [64]. These brain regions showed an increase of adult neurogenesis in animals cognitively stimulated [10]. In our study, the Oct-PCDHs and Oct-pax2/5/8 showed a different level of expression in accordance with the stimulation to which the animals were exposed to: Lower in the control, higher in the tested, and the highest in the wild octopuses. The upregulation of Oct-PCDHs is supported by the key role that these genes play in the nervous system and by the wide expansion of this family gene in the *Octopus* sp. [13,14,15]. In particular, PCDH18 (a and b) genes are involved the essential steps of neurogenesis, such as neuron migration, adhesion, and arborization [65].

**Figure 6:**
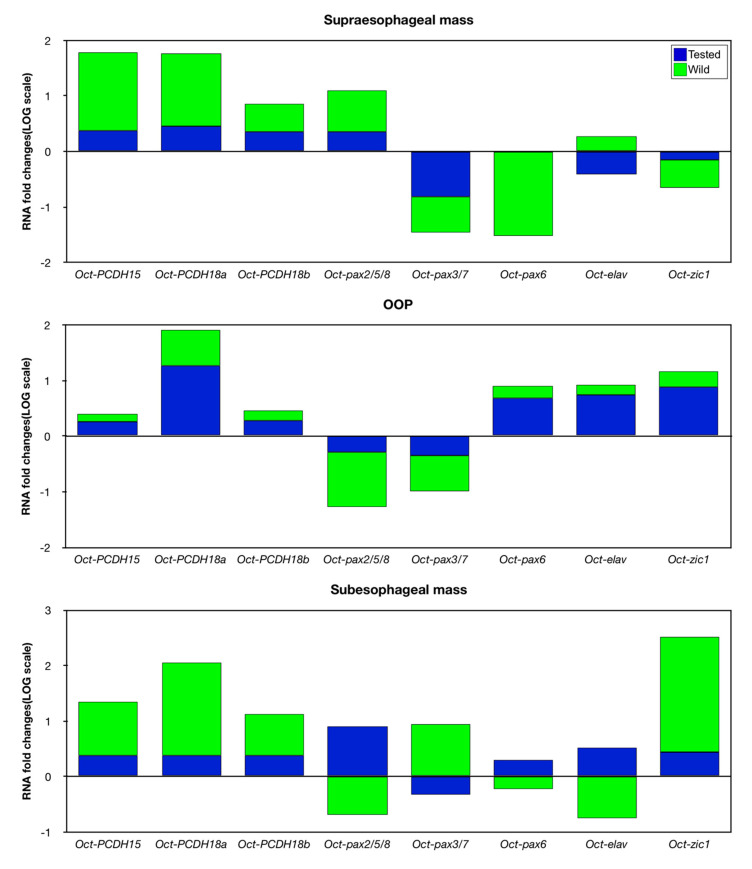
Map of gene expression detected in supraesophageal mass, OOP, and subesophageal mass in *Octopus vulgaris* under different degree of cognitive stimulation. Relative mRNA fold change in gene expression was, in the logarithmic scale, tested and wild octopuses were compared to control (N = 6/group; line set y = 1).

For the other genes considered, we did not observe a similar trend (Figure 4 and Figure 6). Oct-pax6 and Oct-zic1 expression in the tested animals not only show no difference with the control but are even downregulated in wild animals. This finding can be explained by the fact that PAX6 is mainly involved in the neural development of the brachial nervous chord in cephalopods [45]. Otherwise, the ZIC1 gene is involved in the visual system and cerebellar development [66,67].

Furthermore, the Oct-pax3/7 gene is downregulated in both tested and wild animals. In *Sepia officinalis*, this gene and PAX6 gene do not seem involved in neurogenesis of the supraesophageal mass, but they are restricted to the “motor” areas controlling the arms in the subesophageal mass [44]. The Oct-elav gene is instead downregulated in tested animals and upregulated in wild ones. This divergent behavior found in the two stimulated groups (tested and wild) suggests that the Oct-elav gene, as well as Oct-pax 3/7, Oct-pax 6, and Oct-zic 1, are not suitable as adult neurogenesis markers for this brain area.

### 4.3. Lower Motor Centers of The Octopus’s Brain

The lower motor centers of the octopus’s brain, responsible for accurate motor control and coordination, are the anterior part of the subesophageal mass that is involved in the actions of arms and suckers, while the posterior part is involved in the actions of the mantle and viscera [68,69,70]. In these lobes, neuronal proliferative activity was found in the adult octopus [10,12]. In the tested animals, we observed a synergistic effect exerted by an upregulation of all genes considered, except for Oct-pax3/7, suggesting that they are all stimulated by the problem-solving task, which implies complex motor coordination. This pattern is confirmed in wild animals for Oct-PCDHs and Oct-zic1 genes, which showed a higher level of upregulation compared not only with control animals but also with tested octopus.

Interestingly, wild animals showed a significantly higher expression level for the Oct-pax3/7 gene, probably because they face more stimuli coming from the natural environment than the control and tested animals, and it did not appear to be affected by the specific proposed task. On the contrary, Oct-pax2/5/8 and Oct-pax6 genes were upregulated only in the tested octopus, suggesting a specific involvement in the implementation of the nervous network necessary for tuning fine movements, such as those needed to open screw-lid jars [10].

In this brain area, the Oct-elav gene expression also upregulated in tested animals while significantly downexpressed in wild ones. Different from what was observed in supraesophageal mass, in tested octopus, the higher expression of Oct-elav allows us to hypothesize that this gene is specifically involved in the interneuron proliferation. This contributes to the fine motor coordination, supporting our previous finding on adult neurogenesis in the subesophageal mass [10]. Wild octopus showed an opposite trend, with lower Oct-elav gene expression. This latter situation could be due to the multiple options offered by the natural environment in which the animal is not forced to solve a specific problem.

### 4.4. The Multi-Sense Integration Area

The optic and olfactory lobes integrate sensory stimuli detected by sensory organs from the environment while the spine of the peduncle lobe is considered an analog of the vertebrate cerebellum. These lobes are involved in the coding of the visual and chemical inputs as well as the coordination of voluntary movements, such as posture resulting in smooth and balanced muscular activity supporting the neurophysiological bases of octopus’ sophisticated behavior [11,12,59,70,71,72,73,74].

In wild and tested animals, we found upregulation for Oct-PCDHs, Oct-pax6, Oct-elav, and Oct-zic1 genes, suggesting that these genes are related to sensory integration that is strictly connected to the increase of the neuronal structure at adult neurogenesis as previously found in this area [10,12]. On the other hand, the Oct-pax2/5/8 and Oct-pax3/7 genes appeared downregulated in wild animals while no significant differences were found in their expression level in tested octopus, suggesting that in these brain areas, they do not have any role in adult neurogenesis regulation.

## 5. Conclusions

After the discovery that cognitive stimulation positively affects adult neurogenesis in *O. vulgaris’* brain [10], we propose for the first time a link between the process of adult neurogenesis and transcriptomic expression using a large set of genes classified as markers for neurogenetic mechanisms.

With a detailed analysis of expression levels for the selected genes, we found a clearer pair of adult neurogenesis occurring after cognitive stimulation with Oct-PCDHs, which appeared to be upregulated in both tested and wild octopuses. Our study showed that the gene expression level of PCDHs could represent a valuable tool to detect and measure adult neurogenetic processes, including synaptic plasticity. The selected three non-clustered PCDHs are known to be involved during early stages, such as axon outgrowth and path finding [15,21,22].

Furthermore, the next steps could be directed to expand the gene marker panel in order to deeply understand the adult neurogenesis mechanism. We then proposed a novel approach that combining cognitive stimulation and evaluation of the pattern of differential gene expression could give information about remote and proximate causes of animal behavior.

## Supplementary Materials

The following are available online at https://www.mdpi.com/2079-7737/9/8/196/s1: Extra results and discussion; Figure S1. Efficiency of the acclimatization process evaluated as the number of jars opened; Table S1. Primers used in this study; Table S2. mRNA fold change in different brain areas of control animal group (N = 6). Table S3. mRNA fold change in different brain area in different brain area in challenged and wild animals.
