# Supplementary material for: Cognitive Stimulation Induces Differential Gene Expression in Octopus vulgaris: The Key Role of Protocadherins
Source: Biology (Basel). 2020 Jul 30;9(8):196. doi: 10.3390/biology9080196 (PMC7465212; doi:10.3390/biology9080196)
Supplement: Supplementary file 1 [file biology-09-00196-s001.pdf]

Extra results and discussion

Acclimatization experiment

To evaluate the differential gene expression patterns avoiding any kind of stress effects due to the octopus fishing and transport procedures, our first result shows the optimal acclimatization period for an octopus, before any behavioral experiment in captivity [1,2].

We assessed that 14 days of acclimatization, in which the animals were left without any kind of stress, resting and being feed *ad libitum*, is the minimum period that allows octopuses to solve a test appropriately at the very first time (Figure 1).

For the gene expression experiment, *challenged* animals, acclimatized for 14 days, responded appropriately to the problem-solving stimulation opening the two jars at the very first day of training.

The main goal of this work was to find the genetic basis that underlies the adult neurogenesis in *O. vulgaris.* To do that, we used a combination of ethological and genetic approaches to evaluate the gene expression patterns in the specific brain areas previously identifies ad neuronal proliferation sites [3,4].

Figure title and legend

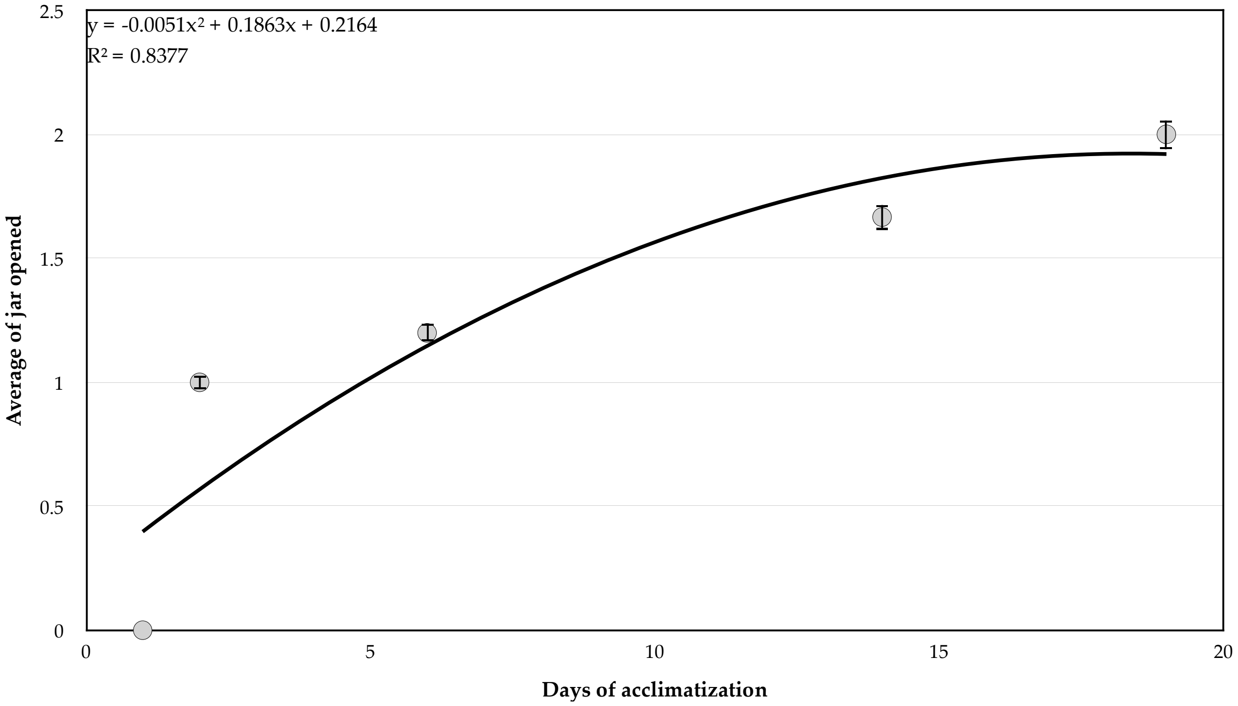

Figure S1 - Efficiency of the acclimatization process evaluated as the number of jar opened.

Tables with titles and legends

Table S1 - Primers used in this study

Genes	Primer sequences (5′→3′)
Ubiquitin (113bp)	F: TCAAAACCGCCAACTTAACC<br>R: CCTTCATTTGGTCCTTCGTC
Oct-PCDH15 (115bp)	F: GACAGAGACAGCAGGCAGAA<br>R: AAACTGGCCGAGAGAAGGAC
Oct-PCDH18a (147bp)	F: AGGCTCGCCTCCTCAAAATG<br>R: GCCGACAGCTTGACAATTGG
Oct-PCDH18b (135bp)	F: GCAAGTTTGGCACGCTTACA<br>R: TCCCTCAGTTGTTGCCTGAC
Oct-pax2/5/8 (103bp)	F: ACAGCTCCGCGTATCTCATG<br>R: TACCTTCGGCTTGGAACCAC
Oct-pax3/7 (136bp)	F: GAAACCTCGCGTTGCTACAC<br>R: ACTAGGTACGGTACTGCGGT
Oct-pax6 (122bp)	F: TTTTGTAAATGGACGGCCGC<br>R: TGCTCACACAACCATTGGAGA
Oct-elav (182bp)	F: GCACGAAATGCATCAACCGATGCGG<br>R: CTGCAGGCCCCT TTAATGCTT TCACT
Oct-zic1 (129bp)	F: TCATGGACACATCACACGGG<br>R: CGTTCGGTTGGGTTCCAAAC

Table S2 - mRNA fold change in different brain areas of control animal group (N=6). Numbers denote the extent of expression of various genes (mean ± SEM) normalized to the expression of housekeeping gene (Ubiquitin) and labeled as $2^{-\Delta Ct}$. Gene expression of subesophageal mass and OOP is compared to supraesophageal mass (t-test, $p<0.05$); **-** indicates genes down-regulated, **+** indicated genes up-regulated, **/** indicate no significant differences. Results with significant differences are highlighted in bold.

Gene	Subesophageal	OOP
Oct-PCDH15	/<br>0.62 ± 0.89	/<br>1.51 ± 1.38
Oct-PCDH18a	+<br>**75.06 ± 0.86**	+<br>**91.46 ± 0.88**
Oct-PCDH18b	+<br>**2.36 ± 0.41**	/<br>1.42 ± 0.37
Oct-pax2/5/8	+<br>**2.62 ± 0.53**	-<br>**0.09 ± 0.83**
Oct-pax3/7	+<br>**1.51 ± 0.30**	/<br>0.98 ± 0.74
Oct-pax6	-<br>**0.17 ± 0.35**	/<br>0.89 ± 0.99
Oct-elav	-<br>**1.57 ± 0.04**	-<br>**1.31 ± 0.23**
Oct-zic1	-<br>**0.39 ± 0.06**	+<br>**1.39 ± 0.23**

Table S3 - mRNA fold change in different brain area in different brain area in challenged and wild animals. Numbers denote the extent of expression of various genes (mean ± SEM, N = 6/group) normalized to the expression of housekeeping gene (Ubiquitin) and labeled as $2^{-\Delta Ct}$. Gene expression of tested and wild animals are compared to control group expression (N=6; t-test, p>0.05); - indicates genes down-regulated, + indicated genes up-regulated, / indicate no significant differences. Results with significant differences are highlighted in bold.

	Supraesophageal		OOP		Subesophageal	
	**Tested**	**Wild**	**Tested**	**Wild**	**Tested**	**Wild**
Oct-PCDH15	+<br>**2.38 ± 0.11**	+<br>**25.28 ± 0.18**	+<br>**1.82 ± 0.07**	/<br>1.37 ± 0.16	+<br>**2.37 ± 0.12**	+<br>**9.48 ± 0.01**
Oct-PCDH18a	+<br>**2.87 ± 0.12**	+<br>**20.53 ± 0.17**	+<br>**18.74 ± 0.10**	+<br>**4.47 ± 0.24**	+<br>**2.46 ± 0.07**	+<br>**45.10 ± 1.10**
Oct-PCDH18b	+<br>**2.25 ± 0.10**	+<br>**3.22 ± 0.19**	+<br>**1.87 ± 0.11**	+<br>**1.56 ± 0.16**	+<br>**2.37 ± 0.34**	+<br>**5.62 ± 0.07**
Oct-pax2/5/8	+<br>**2.24 ± 0.11**	+<br>**5.72 ± 0.10**	/<br>0.53 ± 0.33	-<br>**0.10 ± 0.14**	+<br>**8.01 ± 0.11**	/<br>0.21 ± 0.16
Oct-pax3/7	-<br>**0.15 ± 0.09**	-<br>**0.23 ± 0.12**	/<br>0.45 ± 0.19	/<br>0.23 ± 0.12	-<br>**0.47 ± 0.11**	+<br>**8.78 ± 0.37**
Oct-pax6	/<br>1.00 ± 0.15	-<br>**0.03 ± 0.11**	+<br>**4.82 ± 0.19**	+<br>**1.69 ± 0.18**	+<br>**1.95 ± 0.24**	/<br>0.59 ± 0.16
Oct-elav	-<br>**0.38 ± 0.46**	+<br>**1.87 ± 0.10**	+<br>**5.46 ± 0.26**	/<br>1.57 ± 0.34	+<br>**3.25 ± 0.40**	-<br>**0.18 ± 0.10**
Oct-zic1	/<br>0.72 ± 0.11	-<br>**0.31 ± 0.10**	+<br>**7.80 ± 0.23**	+<br>**1.19 ± 0.31**	+<br>**2.74 ± 0.45**	+<br>**121.10 ± 0.39**

References

1. Malham, S.K.; Runham, N.W.; Secombes, C.J. Lysozyme and antiprotease activity in the lesser octopus *Eledone cirrhosa* (Lam.) (Cephalopoda). *Dev Comp Immunol* **1998**, *22*, 27-37.
2. Walker, J.J.; Longo, N.; Bitterman, M.E. The octopus in the laboratory. Handling, maintenance, training. *Behavior Research Methods & Instrumentation* **1970**, *2*, 15-18, doi:10.3758/BF03205718.
3. Bertapelle, C.; Polese, G.; Di Cosmo, A. Enriched Environment Increases PCNA and PARP1 Levels in *Octopus vulgaris* Central Nervous System: First Evidence of Adult Neurogenesis in Lophotrochozoa. *J. Exp. Zool. B Mol. Dev. Evol.* **2017**, 10.1002/jez.b.22735, doi:10.1002/jez.b.22735.
4. Di Cosmo, A.; Bertapelle, C.; Porcellini, A.; Polese, G. Magnitude Assessment of Adult Neurogenesis in the *Octopus vulgaris* Brain Using a Flow Cytometry-Based Technique. *Frontiers in physiology* **2018**, *9*, 1050, doi:10.3389/fphys.2018.01050.
